# Dehydroepiandrosterone inhibits ADAMTS expression via an ERK-dependent mechanism in chondrocytes

**DOI:** 10.1371/journal.pone.0313560

**Published:** 2024-11-22

**Authors:** Kai Huang, Lin Cheng, Cheng Jiang, Chunwei Zheng, Haili Cai

**Affiliations:** 1 Department of Orthopedic Surgery, Tongde Hospital of Zhejiang Province, Hangzhou, China; 2 Department of Ultrasound, The 903rd Hospital of PLA, Hangzhou, China; University of Vermont College of Medicine, UNITED STATES OF AMERICA

## Abstract

Osteoarthritis (OA) is a joint disease in which cartilage degradation is the hallmark pathological change. In this study, we investigated the anti-osteoarthritic effects of DHEA in rabbit chondrocytes. Polymerase chain reaction was performed to evaluate the expression of a disintegrin and metalloproteinase with thrombospondin motifs (ADAMTS)-4, ADAMTS-5, aggrecan and collagen type 2. In addition, ERK1/2 signaling pathway components were analyzed by Western blotting. In IL-1β-induced chondrocytes, the phosphorylation of ERK1/2 was enhanced, and the downstream catabolic genes, including ADAMTS-4 and ADAMTS-5, were upregulated, while the anabolic genes aggrecan and collagen type 2 were downregulated. DHEA administration restored the IL-1β-induced imbalance in anabolic and catabolic gene expression. In addition, the phosphorylation of ERK1/2 was suppressed by DHEA. Then, PD98059 was used to block the ERK1/2 signaling pathway. The protective effect of DHEA was significantly increased when ERK1/2 signaling was inactivated. DHEA may exert its protective effect by suppressing ADAMTS in an ERK1/2-dependent manner in rabbit chondrocytes.

## Introduction

Osteoarthritis (OA) ranks globally among the 50 most common sequelae associated with diseases and injuries and affects more than 250 million people or 4% of the world population [1]. This condition is progressive and leads to functional declines and the loss in quality of life. OA cannot be completely cured. Current pharmacological OA treatment options are limited to symptomatic relief, but none of these treatments can halt or even reverse the disease in patients [2, 3]. Analgesics, NSAIDs, and intra-articular (IA) viscosupplementation achieve short-term symptomatic pain relief and care of joint functional capacity [4–6]. Glucosamine sulfate has been found to prevent collagen degradation in chondrocytes and to suppress advanced lipoxidation reactions, preventing oxidation [7]. However, the latest guidelines of the American College of Rheumatology does not recommend glucosamine as the initial treatment of OA and does not assure the consistency of glucosamine treatment [8]. IA injections of corticosteroids are recommended for patients with moderate-to-severe pain who do not respond to first-line medication [9]. Currently, there is no cure for OA.

Dehydroepiandrosterone (DHEA) is a 19-carbon steroid hormone synthesized from pregnenolone. DHEA is classified as an adrenal androgen and is rapidly sulfated to its ester form, DHEA-S, which is the predominant form circulating in plasma [10]. DHEA and DHEA-S are the most abundant steroids in human plasma. Because of the levels of these steroids decline with age, the diverse biological functions of DHEA have become an area of intense interest and research.

In animal models, DHEA supplementation has been shown to protect against cartilage damage by exerting anti-inflammatory and anti-degenerative effects on the cartilage. We previously reported that intra-articular (IA) administration of DHEA protects against cartilage degradation and synovial inflammation in rabbits with OA [11]. Our most recent in vivo study showed that DHEA treatment effectively inhibited the progression of existing cartilage degeneration in both knee compartments in moderate OA and in the lateral knee compartment in advanced OA; however, no effects were observed in the medial compartment of the joint in advanced OA, suggesting that the structure-modifying efficacy of DHEA against OA is time-dependent and site-specific [12]. Additionally, a significant portion of DHEA and its sulfate form (DHEAS) is converted into estradiol, which helps protect against OA by maintaining higher estrogen levels [13]. Another key mechanism is that DHEA modulates the Wnt/β-catenin pathway, an important factor in the pathogenesis of OA, which influences cartilage homeostasis and degradation [14]. However, the mechanism by which DHEA provides protection against OA remains not fully understood. ERK1/2 are an important subgroup of MAPKs that play critical roles in processes leading to articular cartilage destruction in OA [15]. A recent ex vivo study using bovine articular cartilage explants showed that ERK1/2 signaling was essential for a disintegrin and metalloproteinase with thrombospondin motifs (ADAMTS)-mediated degradation [16].

Due to the critical role of ERK1/2 signaling in the pathogenesis of OA, in this study, we investigated whether ERK1/2 signaling played a critical role in DHEA-mediated protection against OA, as the connection between DHEA and ERK1/2 signaling has not been reported before.

## Material and methods

### Cell culture and treatment

The study protocol was approved by the Animal Research Committee of Tongde Hospital of Zhejiang Province (Hangzhou, China; Approval no. KTSC2021419). All the authors complied with the ARRIVE guidelines. All methods were performed in accordance with the relevant guidelines and regulations of Tongde Hospital of Zhejiang Province. Ten four-week-old male New Zealand white rabbits were sacrificed by an overdose of sodium pentobarbital administered via the marginal ear vein. Articular cartilage was isolated from the knees under sterile conditions. The cartilage was digested with 0.1% collagenase II for four hours to obtain chondrocytes. The cell suspension was transferred to 75 cm^2^ culture flasks at a density of 10^5^ cells/cm^2^ in 10 ml of DMEM containing 10% FBS and antibiotics (100 units/ml penicillin, 100 lg/ml streptomycin). The chondrocytes were cultured in a 5% CO2 atmosphere at 37.8°C. Confluent primary chondrocytes were passaged at a ratio of 1:3. Chondrocytes at passages two were used for experiments. Adequate measures were taken to minimize animal pain or discomfort.

### Reagents

DHEA, recombinant human IL-1β and collagenase II were obtained from Sigma (St Louis, MO, USA). Recombinant human PD98059 was purchased from Selleck (Houston, TX, USA). The concentrations of PD98059 used were determined according to a previous study [17]. Anti-ADAMTS-4, anti-ADAMTS-5, anti-aggrecan, anti-ARGxx and anti-collagen type 2 antibodies were obtained from Abcam (Cambridge, MA, USA). Dulbecco’s modified Eagle’s medium (DMEM, Hyclone, Logan, Utah, USA), penicillin, streptomycin, fetal bovine serum (FBS) and 0.25% trypsin were obtained from Gibco BRL (Grand Island, NY, USA). DHEA was dissolved in dimethylsulfoxide (DMSO).

### Cell viability assay

Cell viability was measured using the MTT assay. Briefly, cells were seeded in 96-well plates (5 × 10^3^/well) and exposed to different concentrations of DHEA. After a 24-hr incubation period, the cells were washed with phosphate-buffered saline (PBS) and incubated with 5 mg/ml MTT. The solution was removed, and 150 μl of DMSO was added.

### Toluidine blue staining

After adhering to the slide, the cells were washed with 0.02 M PBS. Then, the cells on the microscope slide were fixed with 95% ethanol for 15 s. After air drying, the cells were stained with 0.1% toluidine blue (Leagene, Beijing, China, diluted with 20% alcohol). Then, the excess dye was removed using a paper towel, and the sections were observed using a light microscope (Nikon, Japan).

### Quantitative real-time polymerize chain reaction (PCR)

Total RNA was extracted from cells using TRIzol reagent (Sigma–Aldrich) according to the manufacturer’s instructions. After treatment for 20 min at 37°C with 1 unit of DNase I (Sigma–Aldrich) to prevent genomic DNA contamination, 1 μg of total RNA was reverse transcribed using 1 μg of random hexanucleotide primers (Promega), 0.5 mM dNTPs, and 200 units of Moloney murine leukemia virus reverse transcriptase (Promega) at 37°C for 1 hr in the appropriate buffer. The reaction was stopped by incubation at 70°C for 10 min. Then, quantitative RT-PCR analysis was performed using the iCycler apparatus (Bio-Rad). An iQTM SYBR Green Supermix PCR kit (Bio-Rad) was used for real-time monitoring of amplification (5 ng of template cDNA, 45 cycles: 95°C/10 s, 60°C/25 s) with the primers listed in Table 1. Accurate amplification of the target amplicon was verified by performing a melting curve. Using rabbit 18S rRNA primers (sense 5’-GACGGACCAGAGCGAAAGC-3’; anti-sense 5’-CGCCAGTCGGCATCGTTTATG-3’), parallel amplification of the rabbit 18S transcript (GenBank) was performed to normalize the expression data of the ADAMTS-4, ADAMTS-5, aggrecan and collagen type 2 transcripts. The relative level of expression of these selected genes was calculated for 100 copies of the 18S housekeeping gene according to the formula: n = 10^6^ × 2 ^(Ct 18S _ Ct targeted genes)^.

**Table 1:** Primers used in real-time quantitative RT–PCR amplification.

Gene	Product size (bp)	Primer sequences (5’–3’)	Genbank
ADAMTS-4	128	GAACGGTGGCAAGTATTGTGAGG GGTCGGTTCGGTGGTTGTAG	AF247707
ADAMTS-5	68	CAGTGTTCTCGCTCTTGTGG CTGGGTGCAGGGTTATTGC	AF247708
aggrecan type II collagen	127 123	ATGGCTTCCACCAGTGCG CGGATGCCGTAGGTTCTCA CAGGCAGAGGCAGGAAACTAAC CAGAGGTGTTTGACACGGAGTAG	L38480 D83229.1
18S	119	GACGGACCAGAGCGAAAGC CGCCAGTCGGCATCGTTTATG	EU236696

### Western blot analysis

Cells were washed with ice-cold PBS and harvested. Then, the cells were treated with lysis buffer containing 50 mM Tris-Cl, pH 7.4, 150 mM NaCl, 1 mM EDTA, 1 mM EGTA, 10 lg/ml aprotinin, 10 lg/ml leupeptin, 5 mM phenylmethylsulfonyl fluoride, 1 mM DTT and 1% Triton X-100 for 30 min on ice. The proteins were extracted from cells and transferred to polyvinyl fluoride (PVDF) membranes. The membranes were incubated in Tris-buffered saline with Tween (TBS and 0.1% Tween 20) containing 5% bovine serum albumin overnight at 4°C, followed by incubation with primary antibodies (ERK1/2, p-ERK1/2, collagen type 2, and ARGxx). After being washed, the membranes were incubated for 1 hr at room temperature with HRP-linked secondary antibodies. Finally, the membranes were incubated with the electroencephalographic substrate and exposed to X-ray film (Kodak, Hangzhou, China).

### Statistical analysis

The data are expressed as the means ± standard deviation. The data were analyzed using one-way ANOVA. The Dunnett’s test was used as the posttest for the ANOVA. A value of P < 0.05 was considered to indicate statistical significance.

## Results

### Effects of DHEA on cell viability

**Fig 1:**
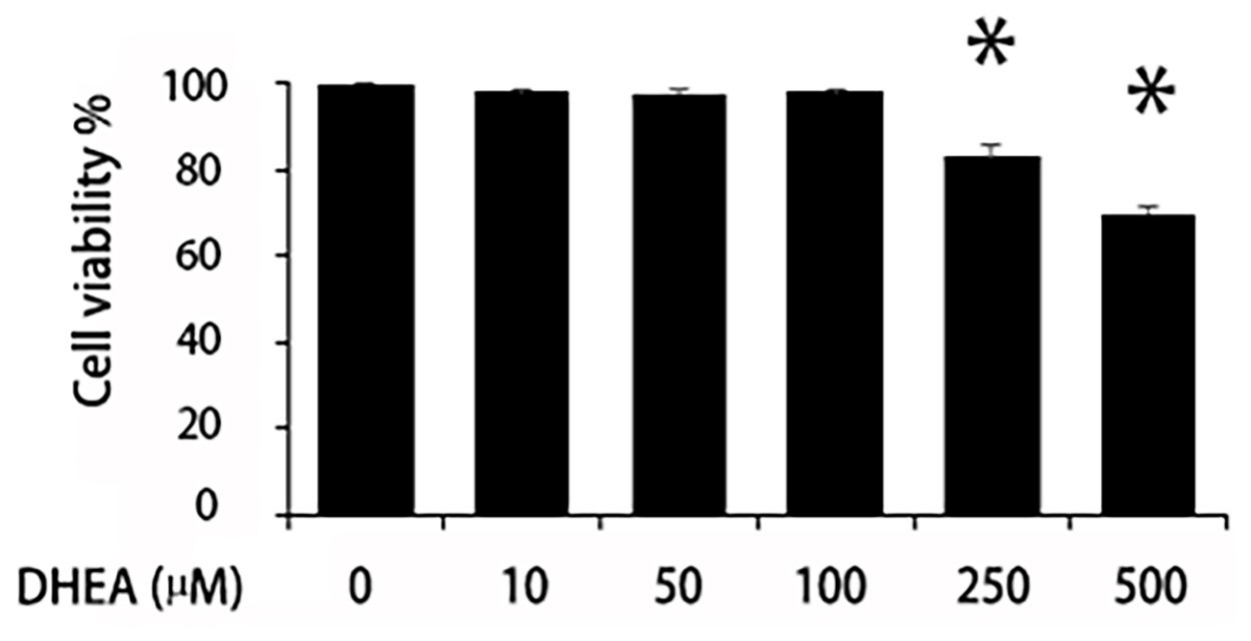
Effects of DHEA on cell viability. Cells were seeded in 96-well plates and cultured with different concentrations of DHEA for 24 hrs, followed by MTT assay analysis. The data presented are the mean ± standard deviation (SD) of three independent experiments.

Cells were exposed to DHEA for 24 hrs, and the results showed that 10 to 100 μM DHEA exerted no adverse effects on the cultured chondrocytes. At 250 and 500 μM DHEA, cell viability decreased markedly (Fig 1). Based on our previous in vivo study showing that 100 μM DHEA efficiently slowed cartilage degradation in a rabbit model of OA [12, 18], we used this concentration (100 μM) in subsequent assessments.

### Effects of DHEA on chondrocyte proteoglycan production

**Fig 2:**
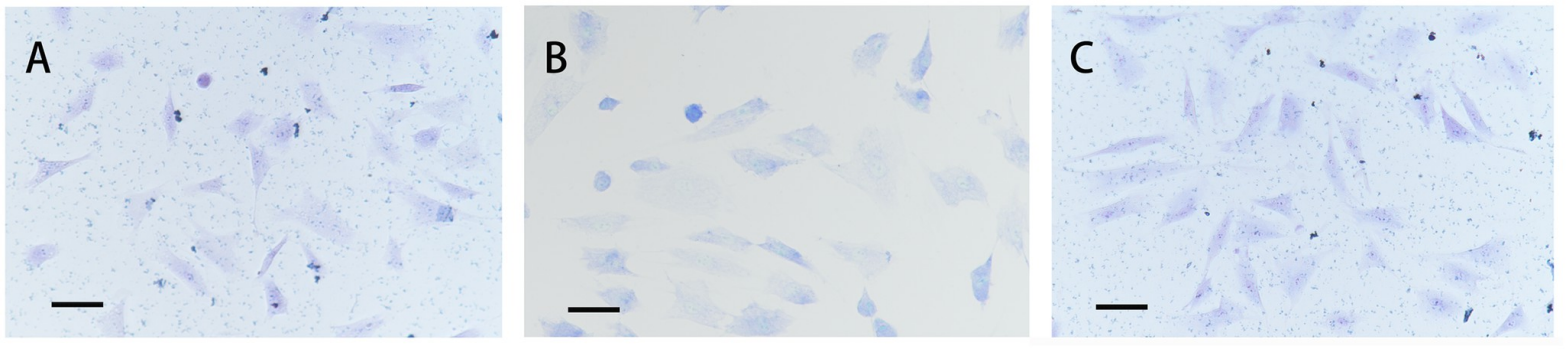
Effects of DHEA on proteoglycan production by chondrocytes. (A) Toluidine blue staining was used to confirm the phenotype of cells harvested from rabbit articular cartilage. (B) IL-1β-stimulated chondrocytes stained with toluidine blue displayed decreased blue staining in the cytoplasm and ECM. (C) IL-1β-stimulated chondrocytes that were pretreated with 100 μM DHEA showed that the blue staining was restored both in the cytoplasm and ECM (magnification, ×200). Scale bar, 25 μm.

Toluidine blue staining was used to confirm the characteristic features of chondrocyte proteoglycans [19]. Compared with IL-1β stimulation, DHEA supplementation obviously enhanced proteoglycan production by chondrocytes, as evidenced by the deeper blue staining in the cytoplasm and ECM (Fig 2).

### Effects of DHEA on ADAMTS-4, ADAMTS-5, aggrecan and collagen type 2 expression in chondrocytes

**Fig 3:**
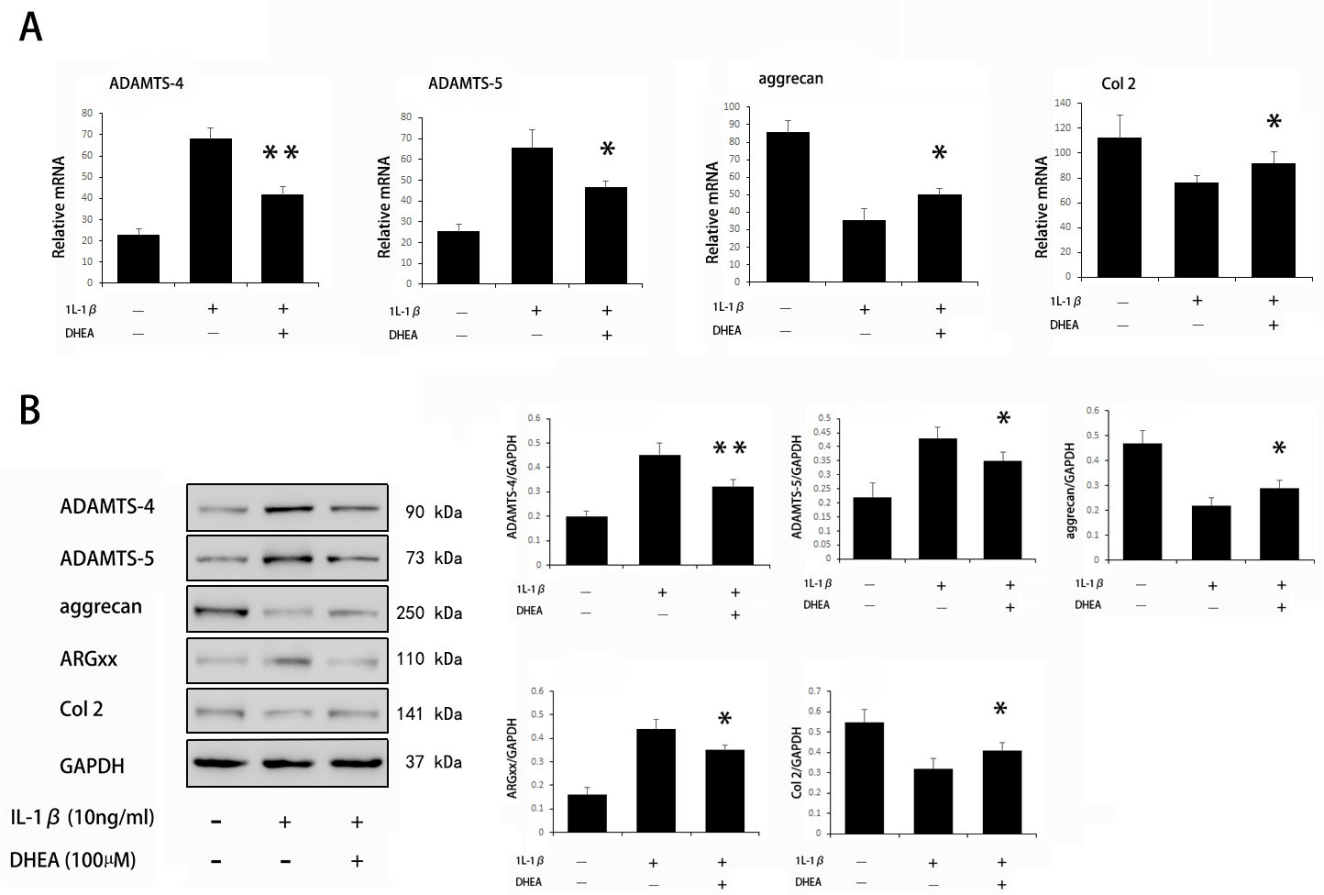
Effect of DHEA on ADAMTS-4, ADAMTS-5, aggrecan and collagen type 2 expression in chondrocytes. Cells were treated with DHEA for 1 hr prior to treatment with IL-1β (10 ng/ml) and were collected after 24 hrs. Quantitative real-time PCR and Western blot analyses were performed to analyze the mRNA and protein expression of target genes, respectively. The data are expressed as the mean ± standard deviation (SD). *P < 0.05 compared with cells stimulated with IL-1β only. **P<0.01 compared with cells stimulated with IL-1β only.

The effects of DHEA on the gene expression of ADAMTS-4 and ADAMTS-5 in IL-1β-induced chondrocytes were assessed by PCR. DHEA significantly inhibited the IL-1β-induced mRNA expression of ADAMTS-4 and ADAMTS-5 while significantly enhancing the expression of ECM-specific genes, including aggrecan and collagen type 2 (Fig 3A). Consistent with the transcriptional level, DHEA also reduced the protein expression of ADAMTS-4 and ADAMTS-5 while upregulating the protein expression of aggrecan and collagen type 2. Notably, DHEA treatment also significantly reduced the protein level of ARGxx, which is a specific aggrecan fragment derived from ADAMTS-4/5 activity [20], suggesting a reduction in ADAMTS-4/5 activity after DHEA administration (Fig 3B).

### Effects of DHEA on ERK1/2 activation in chondrocytes

**Fig 4:**
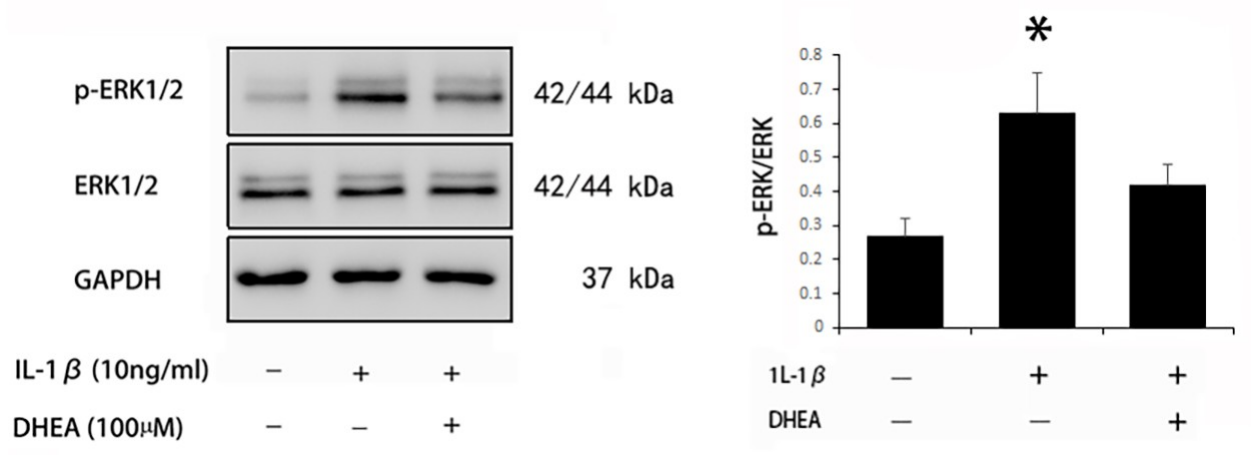
Effect of DHEA on the ERK1/2 signaling pathway in chondrocytes. Western blot analyses were performed to measure the protein expression of p-ERK1/2 and total ERK. The data are expressed as the mean ± standard deviation (SD). *P < 0.05 compared with cells stimulated with IL-1β only.

**Fig 5:**
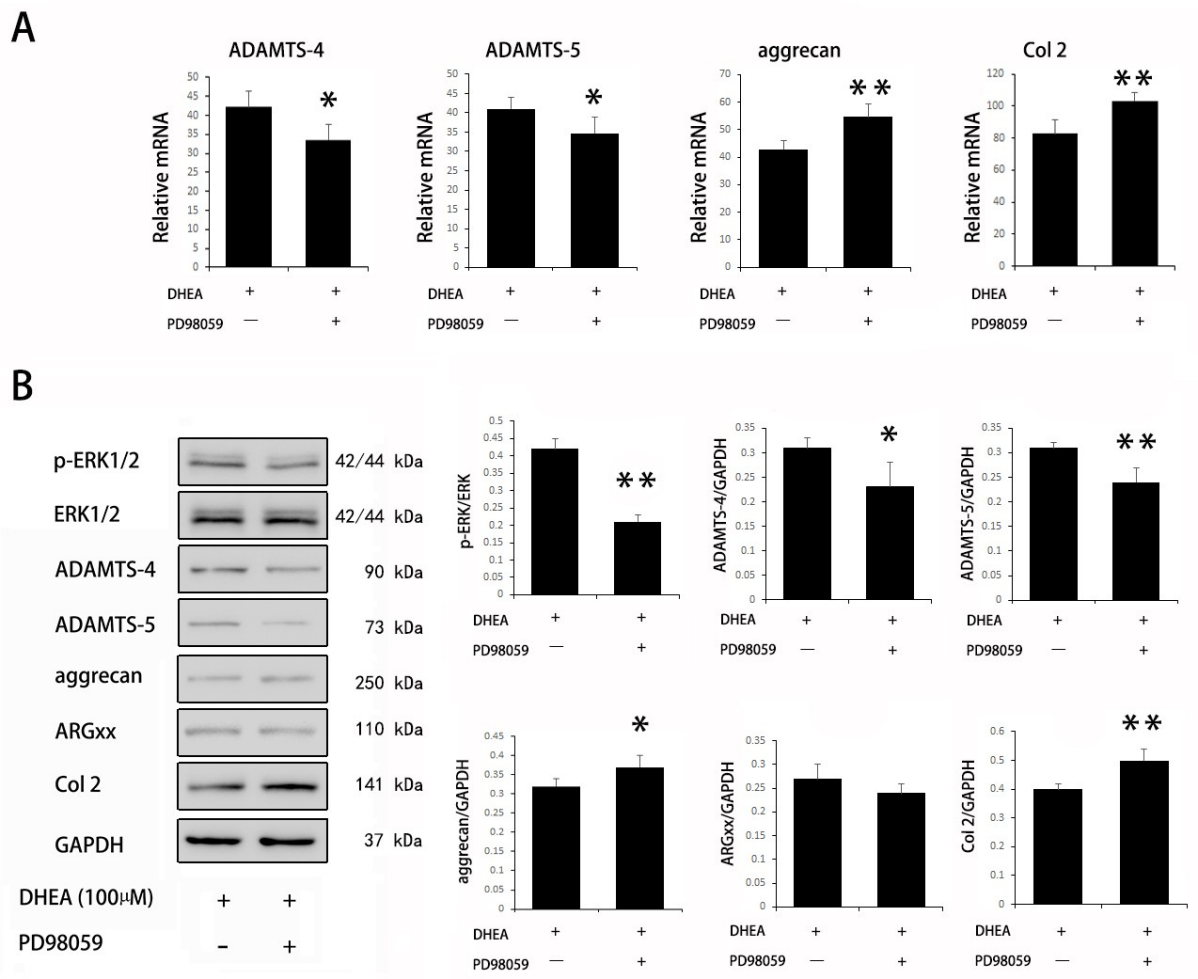
Effect of DHEA on target gene expression after blocking the ERK1/2 pathway in chondrocytes. Cells were treated with DHEA for 1 hr prior to treatment with IL-1β (10 ng/ml) and PD98059 and were collected after 24 hrs. Quantitative real-time PCR and Western blot analyses were performed to measure the mRNA and protein expression of target genes, respectively. The data are expressed as the mean ± standard deviation (SD). *P < 0.05 compared with cells stimulated with IL-1β only. **P<0.01 compared with cells stimulated with IL-1β only.

Activation of the ERK1/2 signaling pathway plays an important role in OA. Previous studies have shown that DHEA can affect the activation of ERK1/2 in other types of cells, including osteoblasts [21] and vascular smooth muscle cells [22]. Thus, in this study, we investigated the effects of DHEA on ERK1/2 in IL-1β-stimulated chondrocytes. The phosphorylation of ERK1/2 was increased by IL-1β, and DHEA significantly inhibited the phosphorylation of ERK1/2 (Fig 4). To further explore whether the suppressive effect of DHEA on ADAMTS-4 and ADAMTS-5 was dependent on ERK1/2 signaling, we observed the effect of DHEA on the expression of the same series of genes in the presence of PD98059, a specific inhibitor of MEK. PCR analysis showed that the mRNA expression of ADAMTS-4 and ADAMTS-5 decreased, while the mRNA expression of aggrecan and collagen type 2 increased with the administration of PD98059 to DHEA-treated chondrocytes (Fig 5A). Western blotting confirmed that PD98059 inhibited the phosphorylation of ERK1/2 in DHEA-treated chondrocytes. In addition, PD98059 induced a consistent trend in the protein expression of the target genes, although the protein level of ARGxx showed no difference (Fig 5B). These results suggest that DHEA suppresses the activation of ERK1/2 signaling in IL-1β-stimulated chondrocytes, and blocking of ERK1/2 signaling can enhance the chondroprotective effect of DHEA, as indicated by decreased mRNA and protein expression of catabolic genes and enhanced mRNA and protein expression of anabolic genes.

## Discussion

DHEA is considered an ‘antidote for aging’ and exerts protective effects against a variety of diseases associated with aging, such as atherosclerosis (AS) [23], osteoporosis (OP) [24] and dementia [25]. OA is also considered an aging-related disorder; however, data concerning the mechanism of DHEA in OA are still limited. Our previous histomorphological study demonstrated the beneficial structure-modifying properties of DHEA on rabbit cartilage in vivo [12]; however, the exact molecular mechanism behind this chondroprotection is still unclear. Recent evidence showed the crucial role of ERK1/2 signaling in the onset of OA and ADAMTS-mediated degradation [16] and the potential of DHEA to inhibit human osteoclastic resorption in an ERK1/2 signaling-dependent manner [21], thus slowing the progress of OP; therefore, we hypothesized that DHEA might slow osteoarthritic cartilage degradation via ERK1/2 signaling in our latest review article [26]. In the current study, we aimed to explore this hypothesis in osteoarthritic chondrocytes.

Aggrecan is the major proteoglycan in cartilage. Aggrecan degradation, which has been attributed to the action of aggrecanases, is an important manifestation of OA [27]. These enzymes have been identified as the proteinases responsible for cleaving the matrix proteoglycan aggrecan at multiple positions; this activity is considered a hallmark of cartilage degradation during inflammatory joint diseases such as OA [28]. Several members of the ADAMTS family of proteins have been shown to cleave aggrecan in vitro at the aggrecanase cleavage site [29–31]. Of these, ADAMTS-4 and ADAMTS-5 are the most efficient aggrecanases and have generally been considered the most likely candidates for a role in the pathogenic mechanisms of OA [32, 33]. Therefore, inhibition of ADAMTS-4/5 might be an effective therapeutic strategy for OA. Indeed, increasing evidence has shown that the deletion of ADAMTS-4/5 provided significant protection against proteoglycan degradation ex vivo and decreased the severity of OA [34–37]. In the current study, we found that DHEA suppressed the mRNA expression of ADAMTS-4 and ADAMTS-5 and enhanced the mRNA expression of aggrecan and collagen type 2 in OA chondrocytes. Additionally, we used an ARGxx antibody, which does not react with intact aggrecan, to detect the aggrecanase-generated aggrecan fragments. The 374ARGS epitope is generated by aggrecanase cleavage at TEGE373-374ARGS [38]; thus, the assay is specific for aggrecanase-mediated aggrecan degradation [39]. Our results showed a reduction in the protein level of ARGxx in the DHEA group, indicating reduced activity of ADAMTS-4/5 in the DHEA group. This finding was consistent with our RT-PCR data.

Many signaling pathways are known to be involved in cartilage degradation in OA; of these, we focused on the ERK1/2 signaling pathway because of its importance in OA progression [40, 41]. For example, the ERK1/2 signaling pathway has been reported to induce ADAMTS-4 and ADAMTS-5 expression, leading to ECM degradation [42]. In the present study, we also demonstrated that IL-1β stimulation induced the phosphorylation of ERK1/2 and enhanced the expression of downstream catabolic genes, including ADAMTS-4 and -5, while DHEA reduced ERK1/2 phosphorylation and rescued ADAMTS-derived aggrecan degradation and proteoglycan loss in chondrocytes. To further explore the role of ERK1/2 signaling in DHEA-mediated protection against OA, we blocked ERK1/2 signaling with the specific inhibitor PD98059 in DHEA-treated chondrocytes. Blocking ERK1/2 signaling significantly enhanced the protective effects of DHEA, as indicated by reduced ADAMTS-4 and -5 expression and increased aggrecan and collagen type 2 expression. These results suggest that the chondroprotective effect of DHEA is partly mediated through the ERK1/2 signaling pathway.

There are some limitations to this study. To address the clinical implications and potential therapeutic strategies for OA, it is essential to consider both biochemical and biomechanical factors contributing to the disease. While the current study focused on the inhibitory effects of dehydroepiandrosterone (DHEA) on IL-1β-induced ADAMTS-4 and ADAMTS-5 expression through the suppression of the ERK1/2 signaling pathway, it remains unclear how DHEA functions under abnormal biomechanical conditions, which are also critical in the onset and progression of OA. Since OA is characterized by impairments in the load-bearing function of articular cartilage, understanding the interplay between biochemical stimuli and biomechanical forces is crucial [43–45]. To address this limitation, future research should focus on chondrocyte metabolism induced by abnormal biomechanics and the effect of DHEA on these chondrocytes. Encouragingly, there are new methods that can achieve these goals. For instance, the Flexcell tension system could exert cyclic mechanical stretch on cultured chondrocytes [46, 47]. Collagen-coated polyacrylamide gels have been used as substrates to culture chondrocytes. The substrate stiffness could be varied by changing the bisacrylamide concentration, thus loading different stresses on chondrocytes [45, 48].

In conclusion, our results demonstrated that DHEA inhibited IL-1β-induced ADAMTS-4 and ADAMTS-5 expression by suppressing the ERK1/2 signaling pathway in rabbit primary chondrocytes. Overall, these results suggest that DHEA may be a potent anti-osteoarthritic agent for the treatment of OA.

## Supporting information

S1 File (PDF)
