# Supplementary material for: Dehydroepiandrosterone inhibits ADAMTS expression via an ERK-dependent mechanism in chondrocytes
Source: PLoS One. 2024 Nov 22;19(11):e0313560. doi: 10.1371/journal.pone.0313560 (PMC11584127; doi:10.1371/journal.pone.0313560)
Supplement: S1 File — (PDF) [file pone.0313560.s001.pdf]

Raw data-PCR

PCR	AD-4		AD-5	aggrecan	col 2
IL-1 -/DHEA -	20.78		26.89	80.34	122.78
IL-1 -/DHEA -	23.12		30.34	89.18	138.71
IL-1 -/DHEA -	27.2		20.78	96.13	99.34
IL-1 -/DHEA -	19.08		25.12	79.56	95.12
IL-1 -/DHEA -	22.14		24.1	82.59	105.6
IL-1 +/DHEA -	66.08		77.13	30.12	67.87
IL-1 +/DHEA -	75.28		64.12	32.68	77.1
IL-1 +/DHEA -	61.98		59.65	45.36	79.13
IL-1 +/DHEA -	67.58		55.86	39.11	80.56
IL-1 +/DHEA -	69.89		70.97	29.19	78.44
IL-1 +/DHEA +	42.88		50.12	47.32	88.34
IL-1 +/DHEA +	43.14		48.45	55.67	79.17
IL-1 +/DHEA +	39.15		43.67	50.19	96.77
IL-1 +/DHEA +	46.88		47.9	49.7	94.3
IL-1 +/DHEA +	36.3		44.14	46.8	102.1

aggrecan	DHEA +/PD98059-	40.2	Col 2	DHEA +/PI	80.2
		44.3			88.1
		39.65			70.34
		42.98			82.1
		47.16			92.84
	DHEA +/PD98059+	55.23		DHEA +/PI	109.3
		57.12			97.26
		60.88			101.87
		49.12			107.44
		51.07			100.91

AD-4	DHEA +/PD98059-	44.76	AD-5	DHEA +/PI	40.09
		40.18			43.81
		39.9			37.71
		48.1			44.29
		38.2			39.15
	DHEA +/PD98059+	30.77		DHEA +/PI	32.19
		35.12			34.33
		29.8			29.51
		40.02			40.33
		32.19			37.02

Raw data-WB

	DHEA(100uM)	DHEA+PD98059	DHEA(100uM)	DHEA+PD98059	DHEA(100uM)	DHEA+PD98059
p-ERK1/2	5589.569	3761.497	5466.447	3560.619	5651.811	3180.619
ERK1/2	9815.104	10268.468	10025.832	10112.711	9887.69	9111.69
ADAMTS-4	3007.276	1733.891	2803.719	1438.062	2436.083	793.305
ADAMTS-5	2257.941	724.991	2356.912	1140.234	2530.376	657.456
aggrecan	1558.012	2284.497	1876.326	3200.811	1614.497	2680.569
ARGxx	3340.619	2538.447	3090.619	2506.205	2729.569	2581.397
Col2	11625.075	17320.64	10081.882	15113.882	9649.154	14152.711
GAPDH	19462.832	19721.175	18137.711	17947.418	17996.368	18787.004

	Control	IL-1β	IL-1β+DHEA	Control	IL-1β	IL-1β+DHEA	Control	IL-1β	IL-1β+DHEA
ADAMTS-4	3758.154	10301.397	5728.276	4057.912	12774.225	5505.276	3810.376	11460.761	5787.861
ADAMTS-5	4307.518	12320.004	5758.175	3440.326	10860.104	4398.983	5336.69	11398.761	7618.397
aggrecan	9879.518	2483.619	3634.74	7660.004	944.891	2593.447	7792.468	2495.497	4718.518
ARGxx	1773.305	5221.326	1629.083	2306.74	7426.64	1383.012	1450.477	6833.276	1105.406
Col2	3883.811	2137.305	4410.74	5088.761	2473.326	4158.74	3906.619	1707.77	3189.397
GAPDH	12892.861	12446.983	13190.175	14650.64	15266.175	13191.69	13089.033	12773.589	13724.468

	Control	IL-1β	IL-1β+DHEA	Control	IL-1β	IL-1β+DHEA	Control	IL-1β	IL-1β+DHEA
p-ERK1/2	2730.548	14632.589	10093.761	5350.619	14420.518	8899.397	5196.033	14130.933	7186.033
ERK1/2	11154.175	10435.104	11036.154	9564.054	10978.933	10990.811	9135.054	9000.347	10374.276
GAPDH	12175.225	11409.376	11045.69	11393.64	13202.418	10071.518	11088.104	11595.468	10507.276

科研课题申报项目实验动物福利伦理审查意见书

尊敬的 黄恺

您提交的编号为 KTSB2021419 的研究方案 软骨基质硬化微环境下 Rho/ROCK 与 ERK1/2 双信号通路“串扰对话”对骨关节炎起病的影响 已经浙江省中医药研究院实验动物福利伦理委员会审查，伦理审查号 KTSC2021419 。

审查结果为 同意申报 ，项目立项后，须再次提交伦理委员会审查。

主任委员签名：

2021 年 7 月 23 日

浙江省中医药研究院实验动物福利伦理委员会
